# Prolonged increase in psychotropic drug use among young women following the COVID-19 pandemic: a French nationwide retrospective study

**DOI:** 10.1186/s12916-024-03496-8

**Published:** 2024-07-02

**Authors:** Antoine Lamer, Chloé Saint-Dizier, Mathieu Levaillant, Jean-François Hamel-Broza, Eiya Ayed, Emmanuel Chazard, Maxime Bubrovszky, Fabien D’Hondt, Michael Génin, Mathilde Horn

**Affiliations:** 1 Fédération Régionale de Recherche en Psychiatrie Et Santé Mentale - F2RSM Psy, Hauts-de-France, Saint-André-Lez-Lille, France; 2 Univ. Lille, CHU Lille, ULR 2694-METRICS: Évaluation des Technologies de santé et des Pratiques médicales, Lille, France; 3 Department of Methodology and Biostatistics, CHU Angers, Angers, France; 4 Inserm, U1085, Irset, équipe ESTER, université d’Angers, faculté de santé, Angers, France; 5 Univ. Lille, Inserm, CHU Lille, U1172 - LilNCog - Lille Neuroscience & Cognition, Lille, 59000 France; 6 Centre National de Ressources Et de Résilience (Cn2r) Lille-Paris, Lille, France

**Keywords:** COVID-19, Mental health, Psychiatry, Pharmacoepidemiology, Psychotropic drugs, Data reuse

## Abstract

**Background:**

The COVID-19 pandemic has had a significant impact on mental health, with evidence suggesting an enduring mental health crisis. Studies worldwide observed increased usage of antidepressants, anxiolytics, and hypnotics during the pandemic, notably among young people and women. However, few studies tracked consumption post-2021. Our study aimed to fill this gap by investigating whether the surge in the number psychotropic drug consumers in France persisted 2 years after the first lockdown, particularly focusing on age and gender differences.

**Methods:**

We conducted a national retrospective observational study based on the French national insurance database. We retrieved all prescriptions of anxiolytics, hypnotics, and antidepressants dispensed in pharmacies in France for the period 2015–2022. We performed interrupted time series analyses based on Poisson models for five age classes (12–18; 19–25; 26–50; 51–75; 76 and more) to assess the trend before lockdown, the gap induced and the change in trend after.

**Results:**

In the overall population, the number of consumers remained constant for antidepressants while it decreased for anxiolytics and hypnotics. Despite this global trend, a long-term increase was observed in the 12–18 and 19–25 groups for the three drug classes. Moreover, for these age classes, the increases were more pronounced for women than men, except for hypnotics where the trends were similar.

**Conclusions:**

The number of people using antidepressants continues to increase more than 2 years after the first lockdown, showing a prolonged effect on mental health. This effect is particularly striking among adolescents and young adults confirming the devastating long-term impact of the pandemic on their mental health.

## Background

**Fig. 1:**
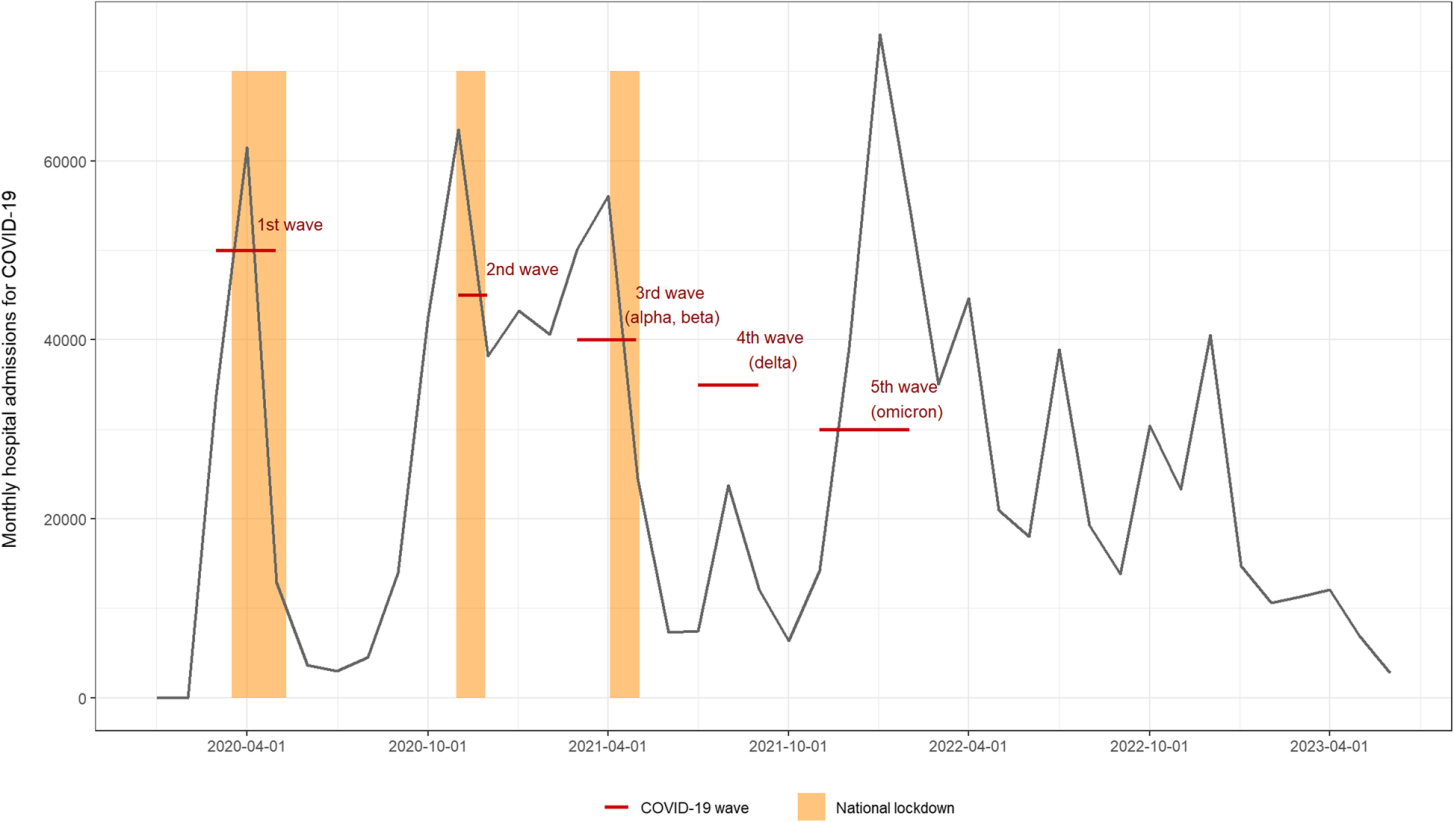
Monthly hospital admissions for COVID-19 in France, COVID-19 waves and national lockdowns

The COVID-19 epidemic emerged in January 2020 in France, with the first wave of hospitalizations occurring in April of that year. Consequently, the first lockdown was established from March 17 to May 11, 2020 (Fig. 1). Subsequently, new variants caused a second wave from September to November 2020, resulting in a new national lockdown between October 30 and November 28, 2020. A third wave with alpha and beta variants occurred from February to May 2021, triggering a third lockdown between April 3 and May 3, 2021. Between July and August 2021, a fourth wave of COVID-19 infections occurred in France, driven by the delta variant. This was followed by a fifth wave between November 2021 and January 2022, caused by the omicron variant. Several social distancing measures, travel restrictions, and vaccine passes were also reimplemented between 2020 and 2022, sometimes on a local scale. In total, the pandemic has claimed the lives of almost 150,000 people in France [1].

The COVID-19 pandemic had a significant impact on healthcare. Hospitals were overwhelmed with coronavirus cases while urban healthcare providers experienced a drop in patient visits, altering their usual activity [2]. In France, the initial lockdown resulted in a decrease in private physician activity, a surge in teleconsultations, a peak in drug provisioning, and a significant decline in vaccination rates [2]. In 2020, hospitalizations were restricted for non-COVID diagnoses, and more individuals passed away at home [3]. The COVID-19 pandemic has also had a significant impact on mental health. Numerous studies conducted during this period have highlighted high prevalence of depression, anxiety, post-traumatic symptoms, and other forms of psychological distress in the general population [4, 5]. Female gender, younger age (≤ 40 years), and chronic and psychiatric illness were identified as risk factors for the severity of psychological distress [5–7]. Beyond its immediate impact, the pandemic’s direct (i.e., infections, hospitalizations, deaths) and indirect (i.e., economic crisis, difficulties in accessing care, isolation) consequences could lead to a prolonged mental health crisis [8–11]. Recommendations have thus been made to monitor the mental health of populations over the next few years [12]. However, longitudinal studies remain rare and difficult to implement.

An interesting approach to studying the effects of the pandemic on mental health is to monitor changes in psychotropic drug consumption [13]. Several studies examined the prescription of psychotropic drugs during the COVID-19 pandemic and observed an increase in the consumption of anxiolytics, hypnotics, and antidepressants in 2020 compared to 2019 [14–19]. In France, similar studies have been conducted using national databases [9, 20, 21], regional [13] or national military samples [22]. As in other countries, these studies revealed an increase in psychotropic drug consumption during the pandemic period, particularly notable among young people [9, 13, 18, 19, 23] and women [13, 19, 23]. Some of these studies have shown an increase in the consumption of psychotropic drugs among young people in the first few months after the beginning of the pandemic [9, 13, 19, 23] in relation to schools closures, reduced outdoor activities, and extensive social media use [24–26, 26, 27]. In line with a recent study of children and adolescents, the present study confirms that the negative effects on young people did not disappear after the pandemic [28]. While those studies assessed psychotropic utilization until 2020 or 2021, no study has explored the evolution of psychotropic consumption in the general population after the end of the sanitary crisis.

To our knowledge, only two studies, conducted among children and young adults, included data until June 2022 and reported an increase in rates of psychiatric disorder diagnoses and psychotropic treatment, particularly pronounced among young aged 12 to 17 years [14, 28, 29]. These findings suggest that the pandemic’s impact on mental health persists. However, data as recent as in this study were not yet analyzed for all the age classes and with an interest in gender effect.

The primary objective of our study was thus to investigate whether the increase in the number of consumers of psychotropic drugs in the general population during the pandemic has been sustained 2 years avec the first year of COVID-19. The secondary objective was to assess whether changes in psychotropics differed according to age and gender. These objectives could be achieved using the French health insurance database covering the whole population.

## Methods

### Database

We conducted a retrospective cohort study using the French health insurance medico-administrative database (SNDS, in French, Système national des données de santé) [30]. The SNDS contains comprehensive individual-level data, including demographics (i.e., age, sex, zip code), and medical consumption for outpatient and inpatient care, such as hospital admissions, consultations, and medications dispensed at community pharmacies. However, it does not document drugs dispensed in hospitals. We followed the reference methodology MR-007 of the Commission nationale de l’informatique et des libertés, which provides a framework for access to data from the SNDS and the conduct of retrospective studies, by healthcare institutions, hospital federations and research teams.

### Study population and data collected

Our study included monthly psychotropic deliveries from January 2015 to December 2022 for patients aged 12 years and older. We identified antidepressants with ATC codes N06A, anxiolytics with ATC code N05B, and hypnotics with ATC code N05C. We collected data on sex and age of patients, categorizing age into five classes, as we had performed in our previous study [9]. The age groups are structured as follows: 12–18 for school-aged children, 19–25 for young adults, 26–50 for adults, and two categories for older individuals, aged 51–75 and over 75. The analysis encompassed the overall population and was further stratified by age and sex.

### Outcome

We first described the annual number of consumers of antidepressants, anxiolytics, and hypnotics, calculating the rate of consumers per 100 inhabitants based on Institut National de la Statistique et Etudes Economiques (INSEE) population data. The annual number of consumers for a drug is defined as the number of patients who receive it at least once during the year, i.e., who have had at least one dispensing of the drug in a community pharmacy.

Then, we analyzed consumption trends by examining the monthly number of individuals receiving at least one box of respectively antidepressants, anxiolytics, and hypnotics.

### Statistical analysis

For each drug and demographic group, we treated the monthly number of individuals receiving at least one box as a time series. Given the seasonal patterns observed in various mental health areas, including antidepressant prescriptions, anxiety, or suicidal attempts [31–33], we decomposed the time series into seasonal parts, trends, and residuals using the moving averages method thanks to the decompose function from the R package “stats.”

To focus on the impact of lockdown, we analyzed the trend part as the monthly number of consumers. A Poisson model was used to model this count. To evaluate the differences in psychotropic drug consumption trends before and after the first lockdown, we used an interrupted time series (ITS) model, characterized by the following equation:

$${y}_{t}\sim P\left({\lambda }_{t}\right)$$

$$\text{log}\left({\lambda }_{t}\right)={\beta }_{0}+{\beta }_{1}t+{\beta }_{2}{D}_{t}+{\beta }_{3}\left(t-{T}_{L}\right){D}_{t}$$

where ${y}_{t}$ denotes the number of individuals for month $t, 1\le t\le 96$ (from January 2015 to December 2022), and ${\lambda }_{t}$ represents the average number of individuals for month $t$. ${D}_{t}$ is an indicator variable that represents the post-lockdown period: it is coded 0 if $t$ is lower than the time of lockdown ${T}_{L}$ (March 2020; *t* = 64), and coded 1 otherwise. The term $\left(t-{T}_{L}\right){D}_{t}$ represents the delay in months since the first lockdown.

We expressed the multiplicative effect of the 1-month increase on the average number of individuals per month for the pre-lockdown and post-lockdown periods, by exponentiating the estimates of ${\beta }_{1}$ and ${\beta }_{1}+{\beta }_{3}$, respectively. At the first month of lockdown, the monthly average number of individuals is multiplied by $\text{exp}\left({\beta }_{2}\right)$ compared to the expected count without interruption. This estimated instantaneous gap corresponds to $\text{exp}\left({\beta }_{0}\right)*\text{exp}\left({\beta }_{2}\right)-\text{exp}\left({\beta }_{0}\right)$ individuals. For the sake of interpretation, we expressed the various multiplicative effects as percentage changes in the average monthly number of individuals, with their 95% confidence interval (CI95). A multiplicative factor *m* greater than 1 traduces a monthly increase of 100*(*m* − 1) percent. For instance, a factor of 1.025 represented a monthly increase of 2.5% in the number of patients. In contrast, a factor *m* lower than 1 corresponds to a decrease of 100*(1 − *m*) percent. Thus, an estimated factor of 0.950 describes a monthly decrease of 5.0% in the number of patients. In the case of constant trends, a multiplicative factor of 1 is found.

We presented the monthly number of patients, the estimated model, and the associated counterfactual representing the expected trend without major events like lockdowns.

## Results

### Influence of the pandemic on psychotropic drug consumption in the overall population

In 2015, the numbers of individuals consuming antidepressants, anxiolytics, and hypnotics per 100 inhabitants were 6.5, 14.3, and 6.1, respectively. In 2022, the rate for antidepressants remained constant (6.5/100 inhabitants), while it decreased for anxiolytics and hypnotics to 13.1 and 4.5 individuals per 100 inhabitants, respectively.

The monthly number of individuals receiving antidepressants was decreasing by 0.021% (CI95 [0.0042; 0.022]) before the lockdown and saw an instant increase of 1.652% (CI95 [1.588; 1.716]) in March 2020 compared to the expected count without the lockdown (representing a difference of 33,222 individuals). After the lockdown, the number increased by 0.313% (CI95 [0.308; 0.317]) per month.

The monthly number of individuals receiving anxiolytics was decreasing by 0.094% (CI95 [0.093; 0.094]) before the lockdown, with an instant increase of 3.701% (CI95 [3.647; 3.755]) in March 2020 compared to the expected count without the lockdown (representing a difference of 114,744 individuals). After the lockdown, this number of individuals continued to decrease by 0.100% (CI95 [0.096; 0.102]) per month.

The monthly number of individuals receiving hypnotics was decreasing by 0.526% (CI95 [0.525; 0.528]) before the lockdown, with an instant increase of 4.000% (CI95 [3.911; 4.085]) in March 2020 compared to the expected count without the lockdown (representing a difference of 61,756 individuals). After the lockdown, this number of individuals continued to decrease by 0.117% (CI95 [0.111; 0.124]) every month.

### Influence of pandemic on consumption of psychotropic drugs for each age group

For the 12–18 age group, the monthly number of individuals receiving antidepressants was multiplied by 1.007 (+ 0.651%) before the first lockdown. At the onset of the lockdown, the number of individuals was multiplied by 1.030 compared to the expected number of individuals without the appearance of the lockdown (which corresponds to a difference of 321 individuals). After the lockdown, the number of individuals was multiplied by 1.026 each month (+ 2.614%).

The increase after the lockdown was lower in other age groups, with respective monthly multiplication factors of 1.016 (+ 1.602%), 1.003 (+ 0.324%), 1.002 (+ 0.245%), and 1.002 (+ 0.202%) for the 19–25, 26–50, 51–75, and 76 + age groups.

Concerning anxiolytics, trends prior to the initial lockdown showed slight fluctuations by age group. The lockdown period saw an increase in all age groups. Post-lockdown, there was an increase in the 12–18 and 19–25 age groups, with an increase of + 0.803% and + 0.485% for the 12–18 and 19–25 groups, respectively. Other age groups experienced declines.

Hypnotic consumption was decreasing in all age groups before the lockdown, with an immediate increase at the lockdown. The most important increase was in the 12–18 group, with a multiplication factor of 1.526, equivalent to 1205 additional patients per month. After the lockdown, this group continued to present the highest increase, with a monthly increase of + 5.06%, signifying a multiplication factor of 1.051. The 19–25 age group showed an increase of + 0.632% every month, denoting a multiplication factor of 1.006. Other age groups experienced declines.

**Fig. 2:**
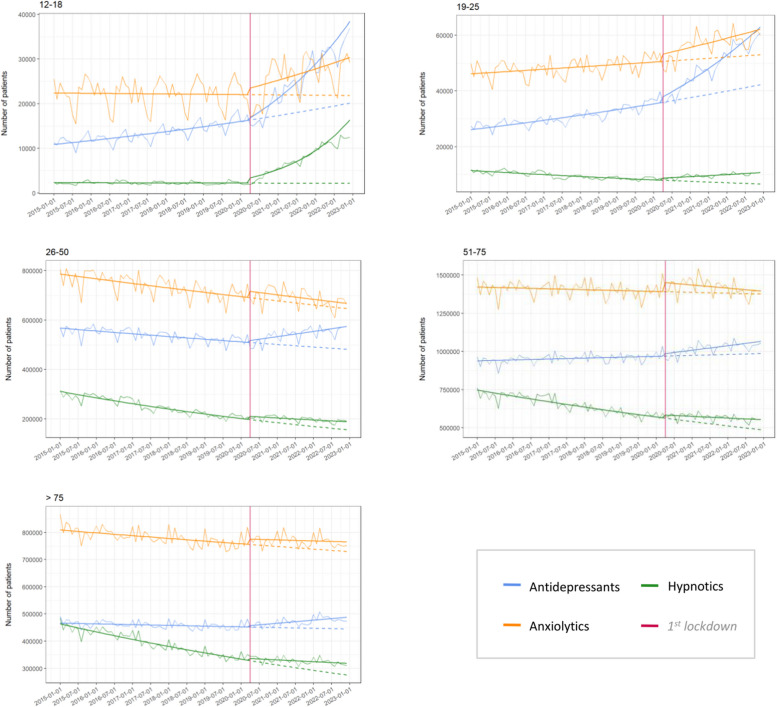
Evolution of monthly drug consumers from 1 January 2015 to 31 December 2022, according to age group and drug considered

All exponential of coefficients and their confident intervals are presented in Table 1. Figure 2 represents the number of individuals receiving antidepressants, anxiolytics, and hypnotics per month, between 2015 and 2022, as well as the estimated models.

**Table 1:** Time effect on the number of consumers per month before and after COVID-19 first lockdown in France, according to drug class and age group

	Trend before COVID-19 exp (B1) [IC95]	Immediate impact exp (B2) [IC95]	Changes in trend exp (B3) [IC95]
**Antidepressant**			
12–18	1.007 [1.006;1.007]	1.030 [1.022; 1.037]	1.020 [1.029; 1.020]
19–25	1.005 [1.005; 1.005]	1.055 [1.050; 1.060]	1.011 [1.011; 1.011]
26–50	0.998 [0.998; 0.998]	1.017 [1.015; 1.018]	1.005 [1.005; 1.005]
51–75	1.001 [1.001; 1.001]	1.017 [1.016; 1.018]	1.002 [1.002; 1.002]
76 and older	1.000 [1.000; 1.000]	1.012 [1.011; 1.014]	1.003 [1.002; 1.003]
**Anxiolytics**			
12–18	1.000 [1.000; 1.000]	1.067 [1.061; 1.073]	1.008 [1.008; 1.009]
19–25	1.002 [1.001; 1.002]	1.052 [1.048; 1.056]	1.003 [1.003; 1.004]
26–50	0.998 [0.998; 0.998]	1.037 [1.036; 1.038]	1.000 [1.000; 1.000]
51–75	1.000 [1.000; 1.000]	1.042 [1.041; 1.043]	0.999 [0.999; 0.999]
76 and older	0.999 [0.999; 0.999]	1.026 [1.025; 1.027]	1.001 [1.001; 1.001]
**Hypnotics**			
12–18	1.000 [0.999; 1.000]	1.526 [1.503; 1.550]	1.051 [1.050; 1.052]
19–25	0.994 [0.994; 0.994]	1.099 [1.088; 1.109]	1.012 [1.012; 1.013]
26–50	0.993 [0.993; 0.993]	1.065 [1.063; 1.067]	1.004 [1.004; 1.004]
51–75	0.996 [0.996; 0.996]	1.036 [1.035; 1.038]	1.003 [1.003; 1.003]
76 and older	0.995 [0.995; 0.995]	1.024 [1.022; 1.026]	1.004 [1.004; 1.004]

### Influence of pandemic on consumption of psychotropic drugs for adolescents and young adults, categorized by sex

**Fig. 3:**
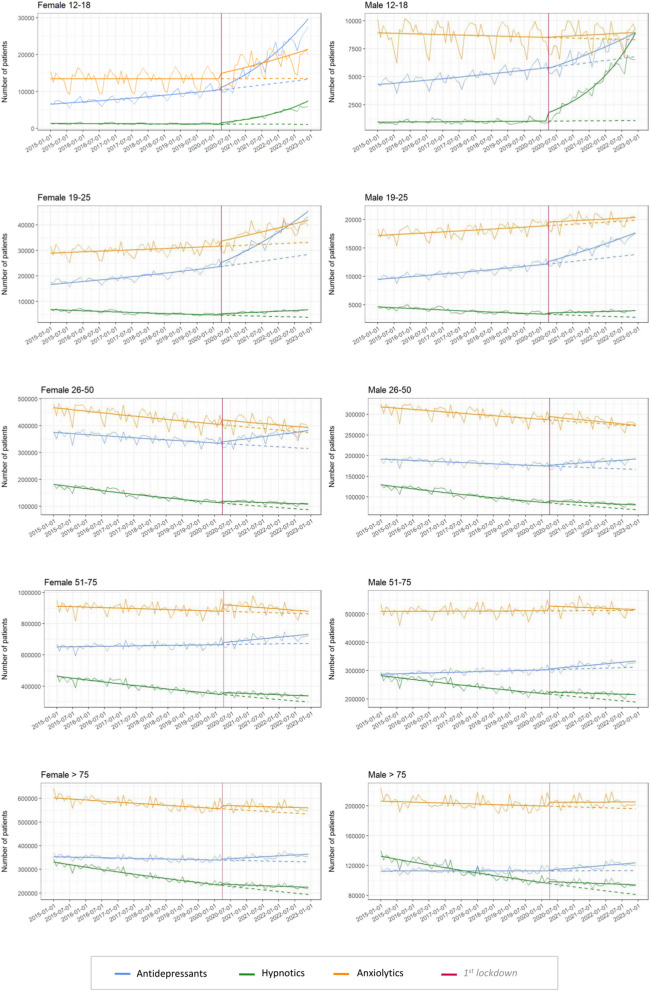
Evolution of monthly drug consumers from 1 January 2015 to 31 December 2022, according to age and sex group and drug considered

Before the first lockdown, the monthly number of females aged 12–18 receiving antidepressants was multiplied by 1.007 (+ 0.75%), with an immediate rise of 384 patients at the onset of the first month of lockdown, which corresponds to a multiplication of 1.059 compared to the expected number of patients without the appearance of the lockdown. After the lockdown, this number of females receiving antidepressants was multiplied by 1.031 each month (+ 3.123%). For males in the same age group, the number of patients was multiplied by 1.014 per month (+ 1.386%). In the 19–25 age group, the number of individuals receiving antidepressants after the lockdown was multiplied by 1.019 and 1.010 each month for females and males, which corresponded to increases of + 1.851% and + 1.052%, respectively. Smaller increases and differences were found in the other groups and are presented in Table 2. Figure 3 represents the number of individuals receiving antidepressants, anxiolytics, and hypnotics per month and by sex, between 2015 and 2022, as well as the estimated models.

**Table 2:** Time effect on the number of consumers per month before and after COVID-19 first lockdown in France, according to drug class, age group, and sex

	Female			Male		
	Trend before COVID-19 exp (B1)	Immediate impact exp (B2)	Changes in trend exp (B3)	Trend before COVID-19 exp (B1)	Immediate impact exp (B2)	Changes in trend exp (B3)
**Antidepressant**						
12–18	1.008	1.059	1.024	1.005	0.987	1.009
19–25	1.006	1.065	1.013	1.004	1.037	1.007
26–50	0.998	1.018	1.006	0.999	1.014	1.004
51–75	1.000	1.020	1.002	1.001	1.011	1.002
76 and older	0.999	1.014	1.003	1.000	1.009	1.003
**Anxiolytics**						
12–18	1.000	1.109	1.011	0.999	1.003	1.002
19–25	1.008	1.317	1.013	1.002	1.034	1.000
26–50	0.998	1.045	1.000	0.998	1.027	0.999
51–75	0.999	1.048	0.999	1.000	1.033	0.999
76 and older	0.999	1.026	1.001	1.000	1.026	1.001
**Hypnotics**						
12–18	0.998	1.332	1.052	1.001	1.745	1.050
19–25	0.994	1.118	1.015	0.995	1.073	1.009
26–50	0.992	1.067	1.005	0.994	1.061	1.003
51–75	0.996	1.036	1.003	0.996	1.037	1.003
76 and older	0.994	1.022	1.004	0.995	1.028	1.004

For anxiolytics, the most important differences between females and males were found for the 12–18 and the 19–25 age groups, with respective increases of + 1.132% versus + 0.151% for 12–18 and + 2.171% versus + 0.121% for 19–25.

For hypnotic consumption, the 12–18 age group reported the most pronounced increases with + 4.993% and + 5.117% for females and males, respectively. In the 19–25 years old group, females presented an increase of + 0.835% while males had an increase of + 0.321%. Coefficients for other age groups are closer between men and women and are presented in Table 2.

## Discussion

This study aimed to examine the effects of the pandemic on psychotropic drug consumers in France 2 years avec the first lockdown. The main findings show that, while the number of people using antidepressants was decreasing before the pandemic, it has steadily increased since the lockdown, with a more pronounced increase among women. When analyzing antidepressant use by age group, we observed a greater increase in individuals under 26 years compared to other age groups. For the other classes of psychotropic drugs, the consumption rapidly declined after the increase associated with the onset of the pandemic, except among adolescents [12–18] and young adults [19–25], where there has been a continuous rise since 2020.

Consistent with Benistand’s recent study, we observed a decrease in the delivery of anxiolytics, hypnotics, and antidepressants in France during the pre-pandemic period. This decrease may be linked to an important prevention campaign conducted in France to limit the prescription of psychotropic drugs [20]. During the pandemic, there was an immediate increase in the number of people using anxiolytics, hypnotics, and antidepressants, which is consistent with the previous studies on consumption during the pandemic [9, 13–20, 22] and could reinforce the reported impact of lockdowns on mental health [6, 8]. Post-pandemic, anxiolytic and hypnotic use decreased, while antidepressant consumption continued to rise. These results underscore the prolonged mental effects of the pandemic, suggesting an increase in the number of people suffering from depression or other mental health conditions that require antidepressants. The decline in anxiolytic and hypnotic use could be attributed to guidelines recommending limited prescription durations for these drugs [34].

Our study reveals the devastating prolonged impact of the pandemic on young people’s mental health. Although the use of antidepressants has increased across all age groups, we observed a particularly important increase among those under 26. Moreover, the number of people in these age groups using anxiolytics and hypnotics, which was slightly increasing before the pandemic, has considerably increased since the beginning of the sanitary crisis. Previous studies have shown an increase in the consumption of psychotropic drugs among young people in the first few months after the beginning of the pandemic [9, 13, 19, 23]. This could be due to a combination of factors, such as school closures, reduced outdoor activities, and extensive social media use [24–27, 35].

The present study confirms that the negative effects on young people did not dissipate 2 years after the first lockdown [28]. Prescribing anxiolytics and antidepressants to adolescents is not a common practice in France, where non-pharmacological treatments are often prioritized for this age group [13]. The important increase in antidepressant and anxiolytic use among adolescents may be related to the severity of their symptoms and/or limited access to psychiatric and psychological support, limiting non-pharmacological treatment options.

The use of psychotropic drugs, specifically antidepressants, is known to be higher among women than men. This trend has been further exacerbated since the beginning of the pandemic. Several hypotheses have been proposed to explain this difference, including a higher prevalence of depression among women, a greater number of women seeking psychiatric help, and increased caregiving responsibilities [16]. A combination of these factors likely contributes to the increased difficulties experienced by women due to the pandemic.

As young people are at high risk of suicidal attempts, a significant concern is the risk of suicidal attempts in this population and particularly for those with depressive disorder [28]. During the lockdown, a French study conducted by Wathelet et al. reported an increased prevalence of suicidal thoughts among students [11]. Although longitudinal studies on suicide attempts in youth during the pandemic are lacking [28], it would be important to assess whether the increase in antidepressant and anxiolytic use is associated with a decrease or increase in suicidal attempts due to more systematic treatment of mental health symptoms or more severe symptoms, respectively.

It could be argued that the increased use of psychotropic drugs indicates an improvement in the detection and treatment of mental health issues in young people. Butt et al. observed an increase in health service utilization related to ADHD after the lockdowns, and Stephenson et al. found a similar increase for depression, suggesting more facilitated access to care [36, 37]. However, several studies suggest the contrary, indicating higher levels of anxiety and depression in children [38, 39]. The increase in demand for medical consultations likely reflects an increase in symptoms.

To the best of our knowledge, this is the first study to assess the evolution of psychotropic drug consumers more than 2 years after the pandemic. Previous studies have evaluated the use of psychotropic medication during the pandemic, revealing a significant increase in the prescription of antidepressants, anxiolytics, and hypnotics likely due to the pandemic’s impact on mental health [9, 20, 40]. Using the psychotropic treatment consumption as an indirect marker of the mental health of French citizens [41], our study further indicates that mental health in France has continued to deteriorate even 2 years after the first lockdown, especially among adolescents and young adults [11]. This study utilized a national database including more than 99% of the French population, providing reliable indicators free from common epidemiological study biases like non-responder or classification bias [9, 20]. Additionally, we considered a large reference period of 5 years before the pandemic to evaluate trends in the use of psychotropic drugs. Last, by stratifying the population by age and sex, we were able to identify more precisely the most severely affected populations.

Our study has some limitations that should be considered. Firstly, the results are based on treatments delivered in pharmacies and it is not possible to determine the number of treatments consumed, home treatment stocks, or prescribed but uncollected medications. Secondly, the prescription reasons are unknown and, in some cases, anxiolytics and antidepressants may have been prescribed for reasons other than anxiety or depression, like epilepsy or pain. Thirdly, despite the use of a national database, we cannot generalize our results to other countries with different pandemic impacts. Fourthly, the statistical model used can be discussed, as it could be considered non-optimal according to the complexity of the prescription rates’ evolution over time. Last, due to the nature of the SNDS data, which are collected for billing purposes, we do not have access to more detailed sociodemographic information, the evaluation of suicidal behavior, and evolutionary data at the diagnostic level.

## Conclusions

More than 2 years after the pandemic, the rising antidepressant use indicates a prolonged effect on mental health, particularly striking among adolescents and young adults. For these groups, anxiolytic and hypnotic consumption has also critically increased since the lockdown, highlighting the devastating pandemic’s prolonged impact on their mental health. Addressing young people’s mental health is a critical public issue and there is an urgent need to strengthen prevention, surveillance, and access to care for this population.

## Data Availability

The data are available only with authorization from the French health insurance medico-administrative database.

## Abbreviations

ATC: Anatomical Therapeutic Chemical code
INSEE: Institut National de la Statistique et Etudes Economiques, French National Institute for Statistics and Economic Studies
ITS: Interrupted time-series
SNDS: Système national des données de santé, French health insurance medico-administrative database
